# Developing WHO guidelines: Time to formally include evidence from mathematical modelling studies

**DOI:** 10.12688/f1000research.12367.2

**Published:** 2018-02-26

**Authors:** Matthias Egger, Leigh Johnson, Christian Althaus, Anna Schöni, Georgia Salanti, Nicola Low, Susan L. Norris

**Affiliations:** 1 Institute of Social and Preventive Medicine (ISPM), University of Bern, Bern, 3012, Switzerland; 2 Centre for Infectious Disease Epidemiology and Research (CIDER), University of Cape Town, Cape Town, 7925, South Africa; 3 World Health Organization, Geneva, Switzerland

**Keywords:** World Health Organization, guidelines, mathematical modelling, study quality, GRADE

## Abstract

In recent years, the number of mathematical modelling studies has increased steeply. Many of the questions addressed in these studies are relevant to the development of World Health Organization (WHO) guidelines, but modelling studies are rarely formally included as part of the body of evidence. An expert consultation hosted by WHO, a survey of modellers and users of modelling studies, and literature reviews informed the development of recommendations on when and how to incorporate the results of modelling studies into WHO guidelines. In this article, we argue that modelling studies should routinely be considered in the process of developing WHO guidelines, but particularly in the evaluation of public health programmes, long-term effectiveness or comparative effectiveness.  There should be a systematic and transparent approach to identifying relevant published models, and to commissioning new models.  We believe that the inclusion of evidence from modelling studies into the Grading of Recommendations Assessment, Development and Evaluation (GRADE) process is possible and desirable, with relatively few adaptations.  No single “one-size-fits-all” approach is appropriate to assess the quality of modelling studies. The concept of the ‘credibility’ of the model, which takes the conceptualization of the problem, model structure, input data, different dimensions of uncertainty, as well as transparency and validation into account, is more appropriate than ‘risk of bias’.

## Introduction

Mathematical models have a long history in public health ^1^. In 1760, Daniel Bernoulli developed a model of smallpox transmission and control. William Hamer published a measles transmission model in 1906 and Ronald Ross a model of malaria transmission in 1908. In recent years, the number of publications related to mathematical modelling has increased steeply. Today, mathematical modelling studies are not restricted to infectious diseases but address a wide range of questions.

The World Health Organization (WHO) provides recommendations on many public health, health system and clinical topics. WHO guidelines are developed using processes and methods that ensure the publication of high-quality recommendations, as outlined in the *WHO Handbook for Guideline Development* ^2^. WHO uses the Grading of Recommendations Assessment, Development and Evaluation (GRADE) approach to rate the certainty of a body of evidence and to produce information that is used by guideline panels to formulate recommendations, based on the balance of benefits and harms and other considerations ^3^.

Many of the questions addressed in mathematical modelling studies are relevant to the development of guidelines. Increasingly, WHO and other guideline developers need to decide whether and how the results of mathematical modelling studies should be included in the evidence base used to develop recommendations. We reviewed the 185 WHO guidelines that were approved by the Guidelines Review Committee from 2007 to 2015: 42 (23%) referred to mathematical modelling studies. However, these studies were rarely formally assessed as part of the body of evidence, and quality criteria for modelling studies were often lacking. A major barrier to the incorporation of evidence from mathematical modelling studies into guidelines is the perceived complexity of the methods used to construct and analyse these studies. At present, there are no widely agreed methods for, or approaches to, the evaluation of the results of mathematical modelling studies, and to their integration with primary data to inform guidelines and recommendations. In April 2016 WHO organized a workshop in Geneva, Switzerland to discuss when and how to incorporate the results of modelling studies into WHO guidelines (see *Acknowledgements* for names of participants). Specifically, the workshop participants discussed the following three questions::

(1) When is it appropriate to consider modelling studies as part of the evidence that supports a guideline?

(2) How should the quality and risk of bias in mathematical modelling studies be assessed?

(3) How can the GRADE approach be adapted to assess the certainty of a body of evidence that includes the results of modelling and to formulate recommendations?

A detailed workshop report is available from WHO ^4^.

The role of modelling in economic evaluation is well recognised in guideline development and at WHO, and was therefore excluded from discussions. At the workshop, we considered the results of a survey of experts (see Box 1) and a rapid literature review (see below). In this paper, which reflects the opinions of the authors but not necessarily that of all workshop participants, we first define models and modelling studies. We then address the three questions outlined above and conclude with some recommendations on the use of evidence from modelling studies in guidelines development.

**Box 1. Web-based expert survey on the role of mathematical modelling in guideline development**

The survey was conducted between March 17 and April 4, 2016. It consisted of 10 questions: four on the characteristics of the respondents, three on the role of mathematical models in guideline development, two questions on quality criteria for mathematical models and one on the challenges in using mathematical modelling in guideline development (see Figure S1). Using snowball sampling, mathematical modellers, epidemiologists, guideline developers and other experts were invited to participate in the survey. A total of 151 individuals from 28 countries and 87 different institutions responded. About half of respondents were modellers, and the other half users of the results from modelling studies. The majority of respondents (58%) had been part of a guideline development group in the past.

Ninety-five percent of respondents answered yes to the question “Should mathematical modelling inform guidance for public health interventions?” and 60% indicated that findings of mathematical modelling studies can sometimes provide the same level of evidence as those of empirical research studies. When asked to list situations in which mathematical modelling could be particularly useful for the development of guidelines, the absence of empirical data on the effectiveness, cost-effectiveness and impact of an intervention, and on the comparative effectiveness of different interventions was most frequently mentioned. We also asked about situations where mathematical modelling studies should not be used or have been inappropriately used in the development of guidelines. Respondents reported that modelling should not be used “to cover up” for the lack of evidence from empirical research, and due emphasis should be given to the uncertainty of model predictions. When asked about the five most important criteria for the quality of reporting of modelling studies, respondents mentioned that the model structure should be clearly described and justified, the important sources of uncertainty reported, and model validity addressed. Assumptions should be clearly stated, justified and discussed and the sources of parameter estimates described. Finally, respondents identified the interpretation of results from modelling studies, the evaluation of their quality and the communication of uncertainty as major challenges in using mathematical modelling in guideline development. These challenges would be best addressed by including at least one modelling expert in guideline development groups.

## What is a mathematical modelling study?

Using a common terminology across different disciplines, for example infectious disease modelling and modelling in chronic disease, will facilitate the assessment, evaluation and comparison of mathematical modelling studies. A broad definition of a *mathematical model* is a “mathematical framework representing variables and their interrelationships to describe observed phenomena or predict future events” ^5^. We make a distinction between a mathematical model and *mathematical modelling studies*, which we define as studies that address defined research questions using mathematical modelling. Mathematical modelling studies typically address complex situations and tend to rely more heavily on assumptions about underlying mathematical structure than on individual-level data. Examples include investigating the potential of HIV testing with immediate antiretroviral therapy to reduce HIV transmission ^6^, or the likely impact of different screening practices on the incidence of cervical cancer ^7^.

*Statistical modelling* is typically concerned with characterizing sources of variation and associations between variables in observed individual-level data drawn from a target population of interest and tends to address questions of a narrower scope than mathematical models. Both statistical and mathematical models can be used to predict future outcomes and to compare different policies. The results from statistical analyses of empirical data often inform mathematical models. Mathematical modelling studies also increasingly integrate statistical models to relate the model output to data.

Workshop participants discussed whether it might be helpful for guideline groups to classify mathematical models in terms of their scope (for example descriptive versus predictive), or technical approach (for example static versus dynamic) ^8^. Discussants argued that a good understanding of what information models can provide and what level of confidence can be placed in that information was more important than a detailed taxonomy of models ^4^.

## Role of mathematical modelling studies in guideline development

Mathematical models typically address questions that cannot easily be answered with randomized controlled trials (RCTs) or observational studies. Table 1 lists specific situations and examples where the results of mathematical modelling are particularly relevant to guideline development, based on the survey, published examples and the Geneva workshop. Mathematical modelling can overcome some of the limitations of results obtained from the carefully controlled settings in which RCTs are typically conducted. First, the main trial results provide an average effect estimate that applies to a specific intervention and study population. Mathematical modelling studies can be used to extrapolate from the results of RCTs to different target groups and settings, to long term outcomes, and to bridge the gap between efficacy and (long-term) effectiveness ^23^. Second, interventions to prevent and control infectious diseases have non-linear effects. RCTs that address short term effects at the individual level might not be suitable for estimating the longer term effects of introducing an intervention, say a vaccine, in a whole population if indirect herd effects influence the incidence of infection and hence the impact of the intervention ^24, 25^. Third, rapid guidance is often needed early in outbreaks or public health emergencies when relevant interventions for prevention or management might simply not have been evaluated. The results of mathematical modelling studies can be used to draft emergency guidelines or to assess the epidemic potential of new outbreaks ^26^.

**Table 1.** Situations in which mathematical modelling studies may be useful for guideline development.

Situation	Examples of relevant mathematical modelling studies
*The long-term effectiveness or cost-* *effectiveness of an intervention is unclear.*	Life time effect on decompensated cirrhosis of obeticholic acid as second- line treatment in primary biliary cholangitis ^9^. Outcomes and costs over 10 years of donepezil treatment in mild to moderately severe Alzheimer’s Disease ^10^. Long-term clinical outcomes, costs and cost-effectiveness of interventions in diabetes mellitus (types 1 and 2) ^11^.
*The outcomes of an intervention in real world,* *routine care settings are unclear.*	Outcomes of medical management of asymptomatic patients with carotid artery stenosis who were excluded from clinical trials ^12^. Effects on blood pressure and cardiovascular risk of variations in patients’ adherence to prescribed antihypertensive drugs ^13^.
*The comparative (relative) effectiveness of* *different interventions overall or in subgroups* *of patients is unclear.*	Comparative effectiveness of different statins and statin doses in patient groups with varying baseline cardiovascular risk ^14^. Relative effect of different strategies of incorporating bevacizumab into platinum-based treatment on survival in ovarian cancer ^15^. Relative real-world drug effectiveness of disease modifying anti-rheumatic drugs (DMARDs) ^16^.
*The overall effects of an intervention at the* *population level, including direct and indirect* *effects, are unknown.*	Effects of different vaccination strategies with serogroup C meningococcal conjugate vaccines on meningococcal carriage and disease ^17^. Public health impact of vaccinating boys and men with a quadrivalent HPV vaccine ^18^. Impact of expanding access to antiretroviral therapy (“treatment as prevention”) on new HIV infections ^19^.
*The population burden of a disease or* *condition is unknown.*	Estimate of the global burden of latent tuberculosis infection ^20^. Burden of healthcare-associated infections on European population health ^21^. Global variation in stroke burden and mortality ^22^.

Source: WHO expert survey and consultation.

The findings of mathematical modelling studies are only as good as the data and assumptions that inform them. Guideline recommendations should therefore not be based on the outputs of models when uncertainty in the empirical data has not been appropriately quantified, when the model makes implausible assumptions or has not been validated adequately, or when the model predictions vary widely over a plausible range of parameter estimates.

## Assessing the quality of a mathematical modelling study: Rapid review

We performed a rapid review of the methodological literature to identify criteria that are proposed to assess the “quality” of mathematical modelling studies (see Table S1 for the detailed search strategy). Specifically, we aimed to identify criteria proposed to assess the quality of single mathematical modelling studies, including best practice standards or criteria for assessing risk of bias or reporting quality and criteria proposed to assess the quality of a body of evidence from mathematical modelling studies. We were also interested in identifying checklists or other instruments developed to assess the quality of mathematical modelling studies. 

**Figure 1.**
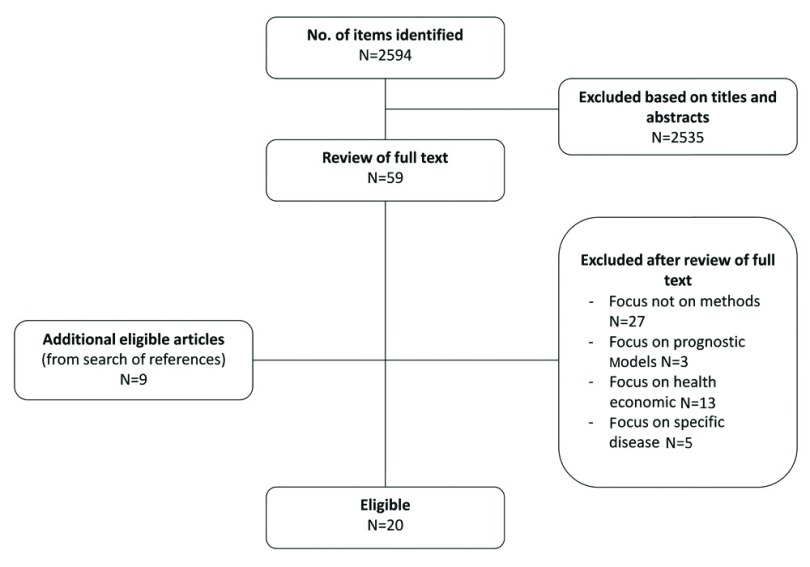
Rapid review of literature on good practice in mathematical modelling: flow of identification of eligible studies.

We identified 20 relevant articles (see Figure 1 for a flow chart of the identification of eligible articles) ^25, 27– 44^. Most gave recommendations for good modelling practice and were compiled by a task force in a consensus process or based on a systematic or narrative review of the literature. The widely cited 2003 paper by Weinstein and colleagues organized 28 recommendations under the headings “structure”, “data”, and “validation” ^31^. A questionnaire or checklist was not included. A subsequent series of seven articles ^25, 38– 42, 44^ by the joint International Society for Pharmacoeconomics and Outcomes Research (ISPOR) and Society for Medical Decision Making (SMDM) task force elaborated upon these recommendations, providing detailed advice on conceptualizing the model, state transition models, discrete event simulations, dynamic transmission models, parameter estimation and uncertainty, and transparency and validation. The 79 recommendations are summarized in the first article of the series ^44^.

We identified four articles ^32, 34, 37, 43^ that present comprehensive frameworks of good modelling practice, with detailed justifications of the items covered and attributes of good practice. They include signalling or helper questions to facilitate the critical appraisal of published modelling studies: the number of questions ranges from 38 in Caro *et al.* ^32^ to 66 questions in Bennett and Manuel ^37^. The four frameworks cover similar territory, including items related to the problem concept, model structure, data sources and synthesis of the evidence, model uncertainty, consistency, transparency and validation ( Table 2). Two of the frameworks include sponsorship and conflicts of interest ^32, 37^.

**Table 2.** Items covered by four published frameworks developed to assess good modelling practice.

Philips 2006 ^34^	Bennett 2012 ^37^	Caro 2014 ^32^	Peñaloza Ramos 2015 ^43^
**Structure** Decision problem/objective Scope/perspective Rationale for structure Structural assumptions Strategies/comparators Model type Time horizon Disease states/pathways Cycle length **Data** Data identification Pre-model data analysis Baseline data Treatment effects Utilities Data incorporation Assessment of uncertainty Methodological Structural Heterogeneity Parameter **Consistency** Internal consistency External consistency	**Structure** Decision problem/objective Scope/perspective Rationale for structure Structural assumptions Strategies/comparators Model type Time horizon Disease states/pathways Cycle length Parsimony **Data** Data identification Data modelling Baseline data Treatment effects Risk factors Data incorporation Assessment of uncertainty Methodological Structural Heterogeneity Parameter **Consistency** Internal consistency External consistency **Validity** Output plausibility Predictive validity **Computer implementation** **Transparency** **Sponsorship**	**RELEVANCE** Population Interventions Outcomes Context **CREDIBILITY** **Validation** External validation Internal verification Face validity **Design** Problem concept Model concept and structure **Data** Process of obtaining and values of inputs **Analysis** Adequacy Uncertainly **Reporting** Adequacy **Interpretation** Balance **Conflict of interest** Potential conflicts and steps taken to address them	**Problem concept** Decision problem Analytical perspective Target population Health outcomes Comparators Time horizon **Model concept** Choice of model type Model structure **Synthesis of evidence** Data sources Utilities Cycle length and half-cycle correction Resources/costs Patient heterogeneity Parameter precision **Model uncertainty** Analyses of uncertainty related to the decision problem Parameter estimation Structural uncertainty Other analyses of uncertainty **Model transparency and validation** Transparency Validation Face validity Internal validity Cross-validation External validity Predictive validity

In a qualitative study Chilcot *et al.* ^27^ performed in-depth interviews with 12 modellers from academic and commercial sectors, and model credibility emerged as the central concern of decision-makers using models. Respondents agreed that developing an understanding of the clinical situation or disease process being investigated is paramount in ensuring model credibility, highlighting the importance of clinical input during the model development process ^27^.

## Model comparisons and modelling consortia

Published mathematical models addressing the same issue may reach contrasting conclusions. In this situation, careful comparison of the models may lead to a deeper understanding of the factors that drive outputs and conclusions. Ideally, the different modelling groups come together to explore the importance of differences in the type and structure of their models, and of the data used to parameterize them ^19, 45, 46^. For example, several groups of modellers have investigated the impact of expanding access to antiretroviral therapy (ART) on new HIV infections. The HIV Modelling Consortium compared the predictions of several mathematical models simulating the same ART intervention programs to determine the extent to which models agree on the epidemiological impact of expanded ART ^19^. The consortium concluded that although models vary substantially in structure, complexity, and parameter choices, all suggested that ART, at high levels of access and with high adherence, has the potential to substantially reduce new HIV infections in the population ^19^. There was broad agreement regarding the short-term epidemiologic impact of ART scale-up, but more variation in longer-term projections and in the efficiency with which treatment can reduce new infections. The impact of ART on HIV incidence long-term is expected to be lower if models: (i) allow for heterogeneity in sexual risk behaviour; (ii) are age-structured; (iii) estimate a low proportion of HIV transmission from individuals not on ART with advanced disease (at low CD4 counts); (iv) are compared to what would be expected in the presence of HIV counselling and testing (compared to no counselling and testing); (v) assume relatively high infectiousness on ART; and (vi) consider drug resistance ^19, 47, 48^.

## Assessing mathematical modelling studies using the GRADE approach

GRADE was conceived with the intention of creating a uniform system to assess a body of evidence to support guideline development in response to a confusing array of different systems in use at that time ^49^. It has since been adopted by over 90 organisations, including WHO. GRADE addresses clinical management questions, including the impact of therapies and diagnostic strategies, diagnostic accuracy questions (i.e., the accuracy of a single diagnostic or screening test), the (cost-) effectiveness and safety of public health interventions, and questions about prognosis.

The GRADE approach encompasses two main considerations: the *degree of certainty* in the evidence used to support a decision and *the strength of the recommendation*. The degree of certainty, i.e., the confidence in or quality of a body of evidence, is rated as “high”, “moderate”, “low”, or “very low” based on an assessment of five dimensions: study limitations (risk of bias), imprecision, inconsistency, indirectness, and publication bias. The initial assessment is based on the study design: RCTs start as high certainty and observational studies as low certainty. Based on the assessments of the five dimensions, RCTs may be down-rated and observational studies up- or down-rated. Judgment is required when assessing the certainty of the evidence, taking into account the number of studies of higher and lower quality and the relative importance of the different dimensions in a given context. The second consideration is the strength of the recommendation, which can be “strong” or “conditional”, for or against an intervention or test, based on the balance of benefits and harms, certainty of the evidence, the relative values of persons affected by the intervention, resource considerations, acceptability and feasibility, among others ^50^. 

We believe that evidence from mathematical modelling studies could be assessed within the GRADE framework and included in the guideline development process. Specifically, guideline groups might include mathematical modelling studies as an additional study category, in addition to the categories of RCTs and observational studies currently defined in GRADE. The dimensions of indirectness, inconsistency, imprecision and publication bias are applicable to mathematical modelling studies, but criteria may need to be adapted. The concept of bias relates to results or inferences from empirical studies, including RCTs and observational studies ^51, 52^ and is too narrow in the context of assessing mathematical modelling studies ^53^. “Credibility”, a term used by ISPOR ^54^, may therefore be more appropriate for modelling studies than “risk of bias”. The assessment of the credibility of a model is informed by a comprehensive quality framework and should cover the conceptualization of the problem, model structure, input data and their risk of bias, different dimensions of uncertainty, as well as transparency and validation ( Table 2). The framework should be tailored to each set of modelling studies by adding or omitting questions and developing review-specific guidance on how to assess each criterion. The certainty of the body of evidence from modelling studies can then be classified as high, moderate, low, or very low. In the evidence-to-decision framework a distinction should be made between *observed outcomes* from empirical studies and *modelled outcomes* from modelling studies (see the Meeting Report ^4^ for an example). 

## Conclusions and recommendations

Based on the discussions and presentations at the workshop in Geneva, the survey and rapid systematic review, we believe a number of conclusions can be formulated.

### When is it appropriate to consider modelling studies as part of the evidence that supports a guideline?

1. The use of modelling studies should routinely be considered in the process of developing WHO guidelines. Findings of mathematical modelling studies can provide important evidence that may be highly relevant. Evidence from modelling studies should be considered specifically in the absence of empirical data directly addressing the question of interest, when modelling based on appropriate indirect evidence may be indicated. Examples for such situations include the evaluation of long-term effectiveness, and the impact of one or several interventions (comparative effectiveness), for example in the context of public health programmes where RCTs are rarely available.

2. Modelling may be more acceptable and more influential in situations where immediate action is called for, but little direct empirical evidence is available, and may arguably be more acceptable in public health than in clinical decision making. In these situations (for example, the HIV, Ebola, or Zika epidemics) funding is also likely to become available to support dedicated modelling studies.

3. The use of evidence from mathematical models should be carefully considered and there should be a systematic and transparent approach to identifying existing models that may be relevant, and to commissioning new models.

### How should the credibility of mathematical modelling studies be assessed?

4. No single “one-size-fits-all” approach is appropriate to assess the quality of modelling studies. Existing frameworks and checklists may be adapted to a set of modelling studies by adding or omitting questions. In some situations, the approach will need to be developed *de novo*.

5. Additional expertise will typically be required in the systematic review groups or guideline development groups to appropriately assess the credibility of modelling studies and interpret their results.

6. The credibility of the models should not be evaluated only by modellers, and not only by modellers involved in the development of these models.

### How can the GRADE approach be adapted to assess a body of evidence that includes the results of modelling and to formulate recommendations?

7. The inclusion of evidence from modelling studies into the GRADE process is possible and desirable, with relatively few adaptations. GRADE is simply rating the certainty of evidence to support a decision and any type of evidence can in principle be included.

8. The certainty of the evidence for modelling studies should be assessed and presented separately in summaries of the evidence (GRADE evidence profiles), and classified as high, moderate, low, or very low certainty.

9. The GRADE dimensions of certainty (imprecision, indirectness, inconsistency and publication bias) and the criteria defined for their assessment are also relevant to modelling studies.

10. For modelling studies, the concept of the ‘credibility’ of the model, which takes the structure of the model, input data, dimensions of uncertainty, as well as transparency and validation into account, is more appropriate than ‘study limitations’ or ‘risk of bias’.

11. When summarizing the evidence, a distinction should be made between observed and modelled outcomes.

12. We propose that within the GRADE system, modelling studies start at low certainty. It should then be possible to increase or decrease the certainty of modelling studies based on a set of criteria. The development of these criteria was beyond the scope of this article; a GRADE working group is addressing this issue ( http://www.gradeworkinggroup.org/).

We look forward to discussing these recommendations with experts and stakeholders and to developing exact procedures and criteria for the assessment of modelling studies and their inclusion in the GRADE process.

## Supplementary material

Table S1. Search strategy in MEDLINE from inception to January 2016 without language restrictions, combining terms for mathematical models with terms for quality assessment and health care decision-making.

Figure S1. Questionnaire of the online survey on the use of mathematical modelling in guidelines for public health decision making.
