# Supplementary material for: Developing WHO guidelines: Time to formally include evidence from mathematical modelling studies
Source: F1000Res. 2018 Feb 26;6:1584. Originally published 2017 Aug 29. [Version 2] doi: 10.12688/f1000research.12367.2 (PMC5829466; doi:10.12688/f1000research.12367.2)
Supplement: Supplementary file 1 [file f1000research-6-15275-s0000.tgz › 833fde43-d743-47dd-a709-36c5b9cf6ad0.docx]

## Table S1. Search strategy in MEDLINE from inception to January 2016 without language restrictions, combining terms for mathematical models with terms for quality assessment and health care decision-making.

| Modelling studies | *MeSH terms* Models, Theoretical/ OR exp Models, Statistical/ OR exp Computer, Simulation/ or exp Markov Chains/ OR *free text words* model?ing.tw. OR (model$ adj3 (stud$ or method$ or process$ or simulation)).tw |
| --- | --- |
|  | AND |
| Quality assessment | *MeSH terms* exp Reproducibility of Results/ OR exp Quality Control/ OR Research Design/ OR *free text words* ((valid$ OR reliab$ OR quality OR accura$) adj2 (result$ OR report$ OR data)).tw. OR ((good or best) adj1 practice$).tw. OR credibility.tw. OR exp Guidelines as Topic/ OR (guideline$ OR checklist$ OR standard$).tw. |
|  | AND |
| Health care decision making | *MeSH terms* Decision Making/ OR Decision Support Techniques/ OR Decision Trees/ OR *free text words* ((mathematical$ or theoretical$) adj3 (structur$ or technique$ or decis$)).tw. Or “public health polic$”.tw. |
|  |  |
| Filter | NOT (Animals / Not humans) |
