# Supplementary material for: Developing WHO guidelines: Time to formally include evidence from mathematical modelling studies
Source: F1000Res. 2018 Feb 26;6:1584. Originally published 2017 Aug 29. [Version 2] doi: 10.12688/f1000research.12367.2 (PMC5829466; doi:10.12688/f1000research.12367.2)

## Figure S1. Questionnaire of online survey on the use of mathematical modelling in guidelines for public health decision making.


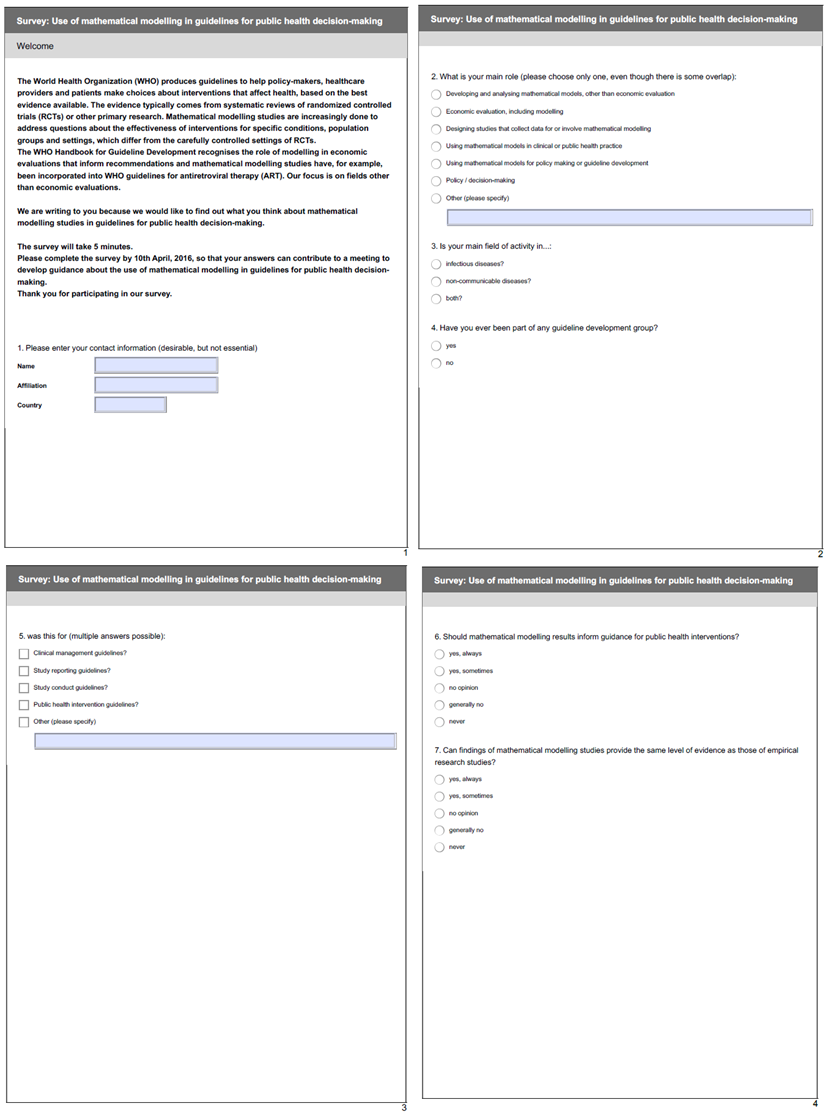


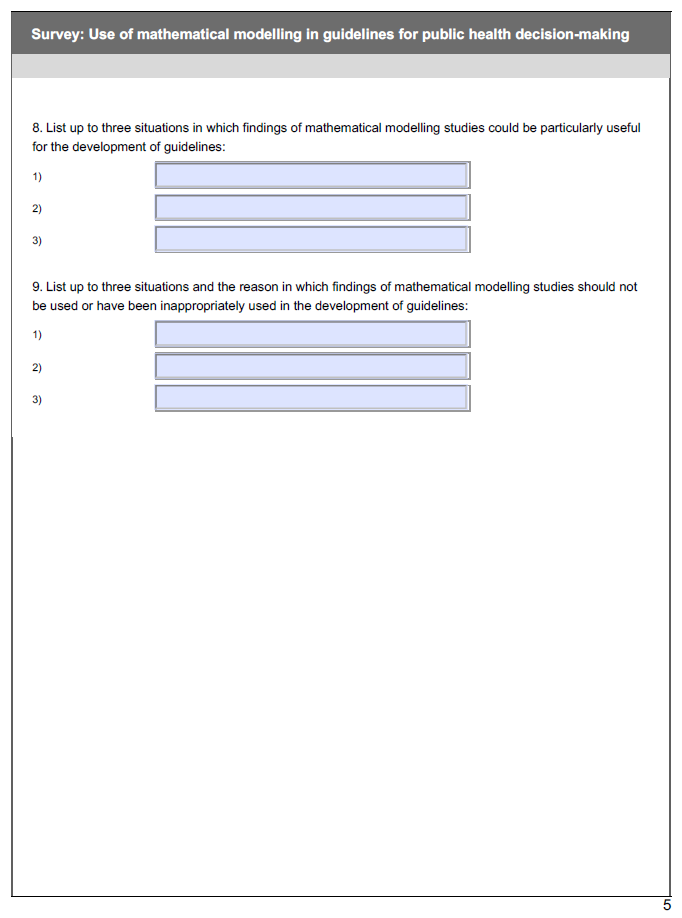

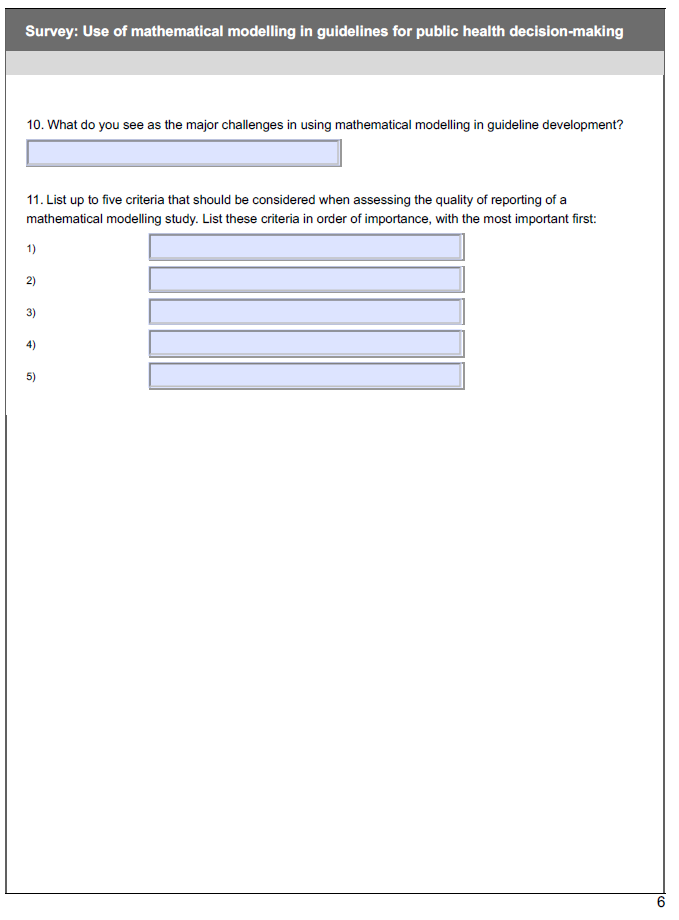

Supplement: Supplementary file 2 [file f1000research-6-15275-s0001.tgz › 254f26d4-eeb3-44e2-ad69-ca3c0f45fbc8.docx]
