# Supplementary material for: Developing WHO guidelines: Time to formally include evidence from mathematical modelling studies
Source: F1000Res. 2018 Feb 26;6:1584. Originally published 2017 Aug 29. [Version 2] doi: 10.12688/f1000research.12367.2 (PMC5829466; doi:10.12688/f1000research.12367.2)
Supplement: Peer review history

# Peer review: Reviewer response for version 2

**DOI:** 10.5256/f1000research.15275.r31178

**Published:** 2018-02-27

**Authors:** Joseph W Hogan

**Affiliations:** 1 Department of Biostatistics and Center for Statistical Sciences, Brown University School of Public Health, Providence, RI, USA

Thanks for the detailed replies; the revision addresses all of my comments.

I confirm that I have read this submission and believe that I have an appropriate level of expertise to confirm that it is of an acceptable scientific standard.

# Peer review: Reviewer response for version 1

**DOI:** 10.5256/f1000research.13392.r25673

**Published:** 2017-11-15

**Authors:** Joseph W Hogan

**Affiliations:** 1 Department of Biostatistics and Center for Statistical Sciences, Brown University School of Public Health, Providence, RI, USA

The authors propose to incorporate findings from mathematical modeling studies into the development of WHO guidelines and other processes related to evaluating and developing public health policies. They argue that evidence from modeling studies should be included in the Grading of Recommendations Assessment, Development and Evaluation (GRADE) process, and make specific recommendations to that effect. The authors argue that model credibility is more appropriate than risk of bias for evaluating strength of evidence generated by modeling studies.  The paper is based on discussions and findings from a meeting of modeling experts in Geneva in 2016; the authors were also participants in the meeting.

 The paper lays out a structured argument for incorporating modeling studies into the evidence base, particularly for formulating WHO recommendations related to treatment of HIV.  The authors start by providing a review of how models are used in various fields, with suggestions about how they can inform guideline development.  They address the question of what constitutes a modeling study.  A comprehensive accounting of published literature on assessment of models is provided.  Finally, they give recommendations for how models can be evaluated using the GRADE approach, with specific conclusions about important issues such as when modeling studies should be used as part of the evidence base and how their credibility should be assessed.

 Given the sweeping variety of models used in published studies about HIV treatments, policies and interventions, the authors are to be applauded for putting forward a framework for having this conversation.  It will promote broader understanding of how models work and how they can be most optimally used for informing treatment guidelines.

 At the crux of their argument is the claim that evidence generated from models should be judged in terms of model credibility rather than on risk of bias.  This argument raises several important issues.  First, what constitutes a mathematical model?  If a model is to be evaluated on its credibility, we need a definition to work from.  Second, what kinds of output from models should be considered evidence, and how should the quality of that evidence be judged and ultimately weighed against or combined with evidence generated from randomized trials and observational cohort data?

**What is a mathematical model?**

 According to the authors, mathematical modeling is “a mathematical framework representing variables and their interrelationships to describe observed phenomena or predict future events.” 

 On its face this is surely true, but for the purpose of understanding whether and how models should add to the evidence base, it’s too broad.  This definition covers a vast assortment of mathematical models, ranging from validated descriptions of natural phenomena (where the mathematical relationships are known and directly observable) to representations of progression from HIV infection to death (comprising known and unknown mathematical components, many of which cannot be directly observed).

 Consider three examples for illustration:

  

1. The mathematical representation of radioactive decay is known and can be written down explicitly.  The model enables accurate and replicable predictions of future observations. 
2. The mathematical model for absorption of a specific drug is typically not known, but empirical studies have shown that it is possible to approximate the systematic variation using nonlinear equations.  These models incorporate known information about physiology and properties of a specific drug, but are necessarily oversimplified representations of drug absorption because there are unobservable characteristics of individuals that affect absorption. The models can be used to make reliable predictions on average, but require unexplained variation to be reflected in terms of prediction intervals. 
3. Now consider a model of the population dynamics of HIV infection and disease progression.  This process also follows a mathematical model, but the model itself is highly complex.  Unlike radioactive decay or rate of drug absorption, the mathematical representations of several components of the underlying processes are essentially unknown.   Moreover, much of the data needed to inform the models are either unobserved (e.g. timing of HIV infection) or only sparsely observed (e.g. individual-level viral load). 

 

 All of these are mathematical models, but definitions must distinguish between them. Otherwise there is an implied equivalence that lends more credibility than is deserved to models that are heavily reliant on unverified assumptions about the mathematical structure underlying the dynamic system being modeled.  A more systematic classification of model types would therefore be helpful.

 While the authors' definition of mathematical model is overly broad, the definition of statistical model, used to contrast with mathematical models, is too narrow.  A statistical model is used to characterize sources of variation in observed data.  It is based on a probabilistic representation of the data generating mechanism, which is itself a mathematical model.  Theory and methods of statistical inference provides a rigorous and transparent set of techniques for parameter estimation, prediction of future outcomes, extrapolation (e.g. for causal inference), and uncertainty quantification. The last of these, uncertainty quantification, is a critical and frequently missing component of predictions based on mathematical models.  

 For the purposes of generating evidence for WHO recommendations, the main difference between a mathematical model and a statistical model is that mathematical models tend to have broader scope and incorporate higher dimensions of complexity, but rely more heavily on assumptions about underlying mathematical structure than on individual-level data.  Statistical models tend to have less mathematical complexity and more narrow scope, and are typically fitted to a single (possibly large) set of observed individual-level data drawn from the target population(s) of interest.  A mathematical structure underlies both statistical and mathematical models, and both can be used for prediction of future outcomes and for causal policy comparisons.  

**Should models be judged on 'risk of bias'?**

 The authors propose that evidence generated from mathematical models should be weighted more heavily toward model credibility than risk of bias.  

 Many mathematical models are over-parameterized relative to the amount of data used to fit them; hence multiple configurations of parameter values can be lead to very similar predictions.  Mathematical models are typically calibrated to observed population-level data (e.g. annual HIV incidence rate for the target population), but the formal rules for doing this seem to vary across application.

 For many consumers of model-based outputs, this is a significant methodologic concern that goes directly to the question of credibility.  If multiple model configurations can generate similar predictions, which configuration is the most credible one?  It seems reasonable that model-based outcomes such as 10-year predictions of HIV incidence need to be evaluated on their own terms.  If coupled with a formal process for back-checking or recalibrating existing models this would surely add value, and would possibly strengthen model identifiability (i.e., provide evidence in favor of one set of model parameters over another).

 A more general justification for incorporating risk of bias into model evaluation can be found in Coveney et al ^1^ (page 4), who provide a general rubric for assessing quality of scientific evidence in the age of big data, emphasizing ‘acceptance of the theory based on concordance between the predictions and the measurements.’   Model calibrations at the time of model fitting partially fulfill this objective, but post-hoc evaluation of model predictions must play an important role in establishing credibility.

 The process of combining and comparing models is highly innovative and likely to have a positive impact on whether the results will be well received.  This kind of cooperation and collaboration, exemplified recently by the Modelling Consortium, is perhaps unique to the mathematical modeling community.  Evidence generated by these kinds of activities can form an important part of the evidence base.

**Summary**

 The authors have provided a thorough case for including results from mathematical modeling into the formal evidence base used for making health recommendations, especially as they relate to HIV.  The paper is based on findings from a recent conference and a comprehensive survey of extant literature. 

 The main critiques are that the definition of mathematical model is far too broad, and that bias (or risk of bias) needs to be incorporated into the evaluation criteria.  Formal methods for uncertainty quantification are critical as well. 

 Mathematical models are prevalent and influential in the HIV literature; hence a discussion about whether and how to place their findings in the broader evidence base is needed and welcome.  This paper provides a necessary starting point.

I confirm that I have read this submission and believe that I have an appropriate level of expertise to confirm that it is of an acceptable scientific standard, however I have significant reservations, as outlined above.

## References

[1] : Big data need big theory too. *Philos Trans A Math Phys Eng Sci*.2016;374(2080) : 10.1098/rsta.2016.0153 10.1098/rsta.2016.0153 27698035PMC5052735

# Response

**Published:** 2018-02-13

**Authors:** Susan Norris

**Affiliations:** World Health Organization, Switzerland

**Joseph W Hogan, Department of Biostatistics and Center for Statistical Sciences, Brown University School of Public Health, Providence, RI, USA**

Approved with Reservations

Authors’ response: Thank you very much for reviewing our paper and making such thoughtful comments. We address your reservations one by one below. We have made changes in the text via tracked changes.

The authors propose to incorporate findings from mathematical modeling studies into the development of WHO guidelines and other processes related to evaluating and developing public health policies. They argue that evidence from modeling studies should be included in the Grading of Recommendations Assessment, Development and Evaluation (GRADE) process, and make specific recommendations to that effect. The authors argue that model credibility is more appropriate than risk of bias for evaluating strength of evidence generated by modeling studies.  The paper is based on discussions and findings from a meeting of modeling experts in Geneva in 2016; the authors were also participants in the meeting.

Authors’ response: First, we would like to stress that our article is an opinion piece, and does not reflect an official position of the World Health Organization or any other body. In the introduction we write: “In this paper, which reflects the opinions of the authors… “. Also, we tried to keep the recommendations fairly general, rather than specific and prescriptive. For example, we refrained from recommending a specific instrument to assess the quality of modeling studies. We conclude by saying that “We look forward to discussing these recommendations with experts and stakeholders and to developing exact procedures and criteria for the assessment of modelling studies and their inclusion in the GRADE process.”

The paper lays out a structured argument for incorporating modeling studies into the evidence base, particularly for formulating WHO recommendations related to treatment of HIV. The authors start by providing a review of how models are used in various fields, with suggestions about how they can inform guideline development.  They address the question of what constitutes a modeling study. A comprehensive accounting of published literature on assessment of models is provided. Finally, they give recommendations for how models can be evaluated using the GRADE approach, with specific conclusions about important issues such as when modeling studies should be used as part of the evidence base and how their credibility should be assessed.

Authors’ response: Thank you, this is a nice outline of the paper.

Given the sweeping variety of models used in published studies about HIV treatments, policies and interventions, the authors are to be applauded for putting forward a framework for having this conversation.  It will promote broader understanding of how models work and how they can be most optimally used for informing treatment guidelines.

Authors’ response: Thank you very much.

At the crux of their argument is the claim that evidence generated from models should be judged in terms of model credibility rather than on risk of bias.  This argument raises several important issues.  First, what constitutes a mathematical model?  If a model is to be evaluated on its credibility, we need a definition to work from.  Second, what kinds of output from models should be considered evidence, and how should the quality of that evidence be judged and ultimately weighed against or combined with evidence generated from randomized trials and observational cohort data?

Authors’ response: We agree that these are central issues. Regarding the outputs from models that should be considered evidence, please note that we distinguish between *mathematical models* and *modelling studies*. The latter address well defined questions and outcomes, such as the impact of HIV testing and immediate antiretroviral therapy on HIV incidence or the impact of different screening strategies on the incidence of cervical cancer. In other words, the modeling outputs that constitute relevant evidence will depend on the question addressed in the modeling studies. In the revised version we write.

GRADE provides a well-defined framework for weighing evidence from randomized trials and observational studies, as discussed in the section on “Assessing mathematical modelling studies using the GRADE approach”. Of note, randomized trials and observational studies are assessed separately. In general, guideline development groups will focus on randomized evidence if such evidence is available from several trial, and only consider observational studies in the absence of substantial randomized evidence. Similarly, evidence from mathematical modelling studies will be considered primarily if other studies cannot answer the question. Statistically combining evidence from different study types is not foreseen in GRADE, and beyond the scope of our article.

**What is a mathematical model?**

According to the authors, mathematical modeling is “a mathematical framework representing variables and their interrelationships to describe observed phenomena or predict future events.” 

On its face this is surely true, but for the purpose of understanding whether and how models should add to the evidence base, it’s too broad. This definition covers a vast assortment of mathematical models, ranging from validated descriptions of natural phenomena (where the mathematical relationships are known and directly observable) to representations of progression from HIV infection to death (comprising known and unknown mathematical components, many of which cannot be directly observed).

Consider three examples for illustration:

1. The mathematical representation of radioactive decay is known and can be written down explicitly.  The model enables accurate and replicable predictions of future observations. 
2. The mathematical model for absorption of a specific drug is typically not known, but empirical studies have shown that it is possible to approximate the systematic variation using nonlinear equations.  These models incorporate known information about physiology and properties of a specific drug, but are necessarily oversimplified representations of drug absorption because there are unobservable characteristics of individuals that affect absorption. The models can be used to make reliable predictions on average, but require unexplained variation to be reflected in terms of prediction intervals. 
3. Now consider a model of the population dynamics of HIV infection and disease progression.  This process also follows a mathematical model, but the model itself is highly complex.  Unlike radioactive decay or rate of drug absorption, the mathematical representations of several components of the underlying processes are essentially unknown.   Moreover, much of the data needed to inform the models are either unobserved (e.g. timing of HIV infection) or only sparsely observed (e.g. individual-level viral load). 

All of these are mathematical models, but definitions must distinguish between them. Otherwise there is an implied equivalence that lends more credibility than is deserved to models that are heavily reliant on unverified assumptions about the mathematical structure underlying the dynamic system being modeled.  A more systematic classification of model types would therefore be helpful.

Authors’ response: Thank you for these three examples. We agree that the definition by Pieter Eykhoff is fairly broad and covers all three categories. However, we feel it is clear from the title and text of our paper that we are primarily concerned with models of the second and third category, i.e. more complex mathematical models that are relevant to WHO guidelines. Within these categories, the level of abstraction and complexity of models and their credibility will of course vary (see also examples in Table 1).

We have now made explicit the distinction that we make between *mathematical models* and *mathematical modelling studies* at the beginning of the section, “What is a mathematical modelling study?” (page 4):

***A broad definition of a mathematical model is a “mathematical framework representing variables and their interrelationships to describe observed phenomena or predict future events”.*** ***^5^*** ***We make a distinction between a mathematical model and mathematical modelling studies, which we define as studies that address defined research questions using mathematical models with a considerable degree of complexity and abstraction.***

At the Geneva workshop participants discussed different types of mathematical models in detail, based on a presentation by one of the authors (CA) on “The anatomy of mathematical modelling studies”. The workshop report and slides and be found at http://apps.who.int/iris/bitstream/10665/258987/1/WHO-HIS-IER-REK-2017.2-eng.pdf. Unfortunately, the link to the report and slides was incorrect in the F1000research paper. CA discussed model dichotomies, based on the book by Ben Bolker (Ecological Models and Data in R, 2008, Princeton University Press), and illustrated these using case studies from the Ebola crisis – see copy of one of his slides at the end of this response. In the discussion, workshop participants argued that guideline groups will often not be able to differentiate between different model dichotomies, and that this is not essential: guideline groups “just have to know what information models can provide and what value can be placed in that information.” However, we recommend that experts in mathematical modelling should support guideline groups (see recommendation 5 on page 9).

We agree with the referee that guideline developers should carefully assess the credibility of models, and that models that “are heavily reliant on unverified assumptions about the mathematical structure underlying the dynamic system” are not credible. Our review of the methodological literature (see Table 2 in the paper) showed that the published frameworks of good modelling practice consistently emphasize the importance of the rationale for the model structure, the structural assumptions and uncertainty, the model transparency and validation etc. See also our recommendations 4, 5 and 6 on p 9.

We added a more explicit reference and the correct link to the Workshop report, (page 3, last line):

***A detailed workshop report is available from WHO ^4^.***

We also expanded the section, “What is a mathematical modelling study” to clarify our view on the need for classifying mathematical modelling studies (page 4, last paragraph):

***Workshop participants discussed whether it might be helpful for guideline groups to classify mathematical models in terms of their scope (for example descriptive versus predictive) or technical approach (for example static versus dynamic) ^8^. Discussants argued that a good understanding of what information models can provide and what level of confidence can be placed in that information was more important than a taxonomy of models ^4^.***

While the authors' definition of mathematical model is overly broad, the definition of statistical model, used to contrast with mathematical models, is too narrow.  A statistical model is used to characterize sources of variation in observed data.  It is based on a probabilistic representation of the data generating mechanism, which is itself a mathematical model.  Theory and methods of statistical inference provides a rigorous and transparent set of techniques for parameter estimation, prediction of future outcomes, extrapolation (e.g. for causal inference), and uncertainty quantification. The last of these, uncertainty quantification, is a critical and frequently missing component of predictions based on mathematical models.

Authors’ response: We agree with the reviewer’s comment about assessing uncertainty in mathematical models and state this explicitly (page 5, paragraph 2).   

For the purposes of generating evidence for WHO recommendations, the main difference between a mathematical model and a statistical model is that mathematical models tend to have broader scope and incorporate higher dimensions of complexity, but rely more heavily on assumptions about underlying mathematical structure than on individual-level data.  Statistical models tend to have less mathematical complexity and more narrow scope, and are typically fitted to a single (possibly large) set of observed individual-level data drawn from the target population(s) of interest.  A mathematical structure underlies both statistical and mathematical models, and both can be used for prediction of future outcomes and for causal policy comparisons.  

Authors’ response: We are grateful to the referee for this insightful and well-phrased comment about the relevance of the terms ‘statistical modelling’ and ‘mathematical modelling’ to WHO guidelines. We have taken the liberty of paraphrasing the comment to  revise this section (page 4) as follows:

***Mathematical modelling studies may address complex situations and tend to rely more heavily on assumptions about underlying mathematical structure than on individual-level data. Examples include investigating the potential of HIV testing with immediate antiretroviral therapy to reduce HIV transmission*** ***^6^*** ***,*** ***or the likely impact of different screening practices on the incidence of cervical cancer*** ***^7^*** ***.***

***Statistical modelling is typically concerned with characterizing sources of variation and associations between variables in observed*** ***individual-level data drawn from a target population of interest*** ***. Statistical models tend to be narrower in scope than mathematical models. Both*** ***statistical and mathematical models*** ***can be used to predict future outcomes and to compare different policies. The results from statistical analyses of empirical data often inform mathematical models. Mathematical modelling studies also increasingly integrate statistical models to relate the model output to data.***

**Should models be judged on 'risk of bias'?**

The authors propose that evidence generated from mathematical models should be weighted more heavily toward model credibility than risk of bias.  

Authors’ response: Yes, we believe that the concept of model credibility is more useful than the more narrow concept of risk of bias (RoB). However, we think there is a mis-understanding here: the assessment RoB of specific studies also has a role.

The RoB concept is widely used in the context of randomized controlled trials and observational studies that aim to make causal inference, and dedicated “RoB tools” have been developed to assess the risk of bias of studies included in systematic reviews (see references 1,2 below and www.riskofbias.info). These tools are based on relatively few well-defined biases. In the case of randomized trials they include selection bias, performance bias, detection bias, attrition bias and reporting bias (1).

In the context of mathematical modelling studies, the risk of bias of empirical studies contributing parameter estimates is important and should be considered, for example in sensitivity analyses. On the other hand, many other and additional aspects are important when assessing the trustworthiness or credibility of mathematical models. These aspects are listed in Table 2, based on a review of published frameworks developed to assess good modelling practice. Please note that we use the term credibility as applied by the International Society for Pharmacoeconomics and Outcomes Research (ISPOR) to assessment of studies for decision making (3).

These frameworks include assessments of the quality of the data used to parameterize a model. For example, Bennett and Manuel (4) and Philips et al (5) include several questions to that effect:

- Where choices have been made between data sources, are these justified appropriately?
- Where data from different sources are pooled, is this done in a way that the uncertainty relating to their precision and possible heterogeneity is adequately reflected?
- Has the quality of the data been assessed appropriately?

The questionnaire proposed by Caro et al (6) asks

- Are the data used in populating the model suitable for your decision problem?
- All things considered, do you agree with the values used for the inputs?

 

Similarly, the framework of Ramos and colleagues (7) includes the following questions:

- Have transition probabilities and intervention effects been derived from representative data sources for the decision problem?
- Have parameters relating to the effectiveness of interventions derived from observational studies been controlled for confounding?

 

We have clarified our position and the role of RoB assessments as follows on page 8:

***The concept of bias relates to results or inferences from empirical studies, including randomized controlled trials and observational studies ^38,39^ and is too narrow in the context of assessing mathematical modelling studies. ^40^*** ***“Credibility”, a term used by ISPOR,*** ***^41^*** ***may therefore be more appropriate for modelling studies than “risk of bias”.*** ***The assessment of the credibility of a model is informed by a comprehensive quality framework and should cover the conceptualization of the problem, model structure, the input data and their risk of bias, different dimensions of uncertainty, as well as transparency and validation (Table 2).***

1. Higgins JP, Altman DG, Gotzsche PC, et al. The Cochrane Collaboration’s tool for assessing risk of bias in randomised trials. BMJ 2011; 343:d5928.

2. Sterne JA, Hernán MA, Reeves BC, et al. ROBINS-I: A tool for assessing risk of bias in non-randomised studies of interventions. BMJ 2016; 355:i4919.

3. Aronson N, Grant MD. Tools for health care decision making: observational studies, modeling studies, and network meta-analyses. Value Health 2014; 17:141–142.

4. Bennett C, Manuel DG: Reporting guidelines for modelling studies. BMC Med Res Methodol. 2012; 12: 168.

5. Philips Z, Bojke L, Sculpher M, et al.: Good practice guidelines for decision- analytic modelling in health technology assessment: a review and consolidation of quality assessment. Pharmacoeconomics. 2006; 24(4): 355–71.

6. Jaime Caro J, Eddy DM, Kan H, et al.: Questionnaire to assess relevance and credibility of modeling studies for informing health care decision making: An ISPOR-AMCP-NPC good practice task force report. Value Health. 2014; 17(2): 174–82.

7. Ramos MC, Barton P, Jowett S, et al.: A Systematic Review of Research Guidelines in Decision-Analytic Modeling. Value Health. 2015; 18(4): 512–29.

Many mathematical models are over-parameterized relative to the amount of data used to fit them; hence multiple configurations of parameter values can be lead to very similar predictions.  Mathematical models are typically calibrated to observed population-level data (e.g. annual HIV incidence rate for the target population), but the formal rules for doing this seem to vary across application.

Authors’ response: We agree – model concept, structure and parsimony are important elements when evaluating the credibility of mathematical models. Validation and predictive validity are also very important – again see Table 2.

For many consumers of model-based outputs, this is a significant methodologic concern that goes directly to the question of credibility.  If multiple model configurations can generate similar predictions, which configuration is the most credible one?  It seems reasonable that model-based outcomes such as 10-year predictions of HIV incidence need to be evaluated on their own terms.  If coupled with a formal process for back-checking or recalibrating existing models this would surely add value, and would possibly strengthen model identifiability (i.e., provide evidence in favor of one set of model parameters over another).

Authors’ response: We agree and, again, believe that these issues are covered by the frameworks we present in Table 2.

A more general justification for incorporating risk of bias into model evaluation can be found in Coveney et al ^1^ (page 4), who provide a general rubric for assessing quality of scientific evidence in the age of big data, emphasizing ‘acceptance of the theory based on concordance between the predictions and the measurements.’   Model calibrations at the time of model fitting partially fulfill this objective, but post-hoc evaluation of model predictions must play an important role in establishing credibility.

Authors’ response: The timely piece by Coveney and Dougherty is really a critique of “’blind’ big data projects” and a plea for “the elucidation of the multiscale and stochastic processes controlling the behaviour of complex systems, including those of life, medicine and healthcare.” We could not agree more and argue that insights from the latter (mathematical models) should inform the development of WHO guidelines.

The process of combining and comparing models is highly innovative and likely to have a positive impact on whether the results will be well received. This kind of cooperation and collaboration, exemplified recently by the Modelling Consortium, is perhaps unique to the mathematical modeling community.  Evidence generated by these kinds of activities can form an important part of the evidence base.

Authors’ response: We completely agree and have stressed this point in our paper.

**Summary**

The authors have provided a thorough case for including results from mathematical modeling into the formal evidence base used for making health recommendations, especially as they relate to HIV.  The paper is based on findings from a recent conference and a comprehensive survey of extant literature. 

The main critiques are that the definition of mathematical model is far too broad, and that bias (or risk of bias) needs to be incorporated into the evaluation criteria.  Formal methods for uncertainty quantification are critical as well. 

Authors’ response: Thank you again for reviewing our paper. See our responses above. We hope that based on our responses and the changes made in the manuscript you will be able to approve our contribution.

Mathematical models are prevalent and influential in the HIV literature; hence a discussion about whether and how to place their findings in the broader evidence base is needed and welcome.  This paper provides a necessary starting point.

# Peer review: Reviewer response for version 1

**DOI:** 10.5256/f1000research.13392.r25463

**Published:** 2017-09-20

**Authors:** Wilma A. Stolk

**Affiliations:** 1 Erasmus MC, Department of Public Health, University Medical Center Rotterdam, Rotterdam, The Netherlands

In this opinion article, the authors discuss when and how to incorporate the results of modelling studies into WHO guidelines, by addressing three questions: (1) When is it appropriate to consider modelling studies as part of the evidence that supports a guideline? (2) How should the quality and risk of bias in mathematical modelling studies be assessed? (3) How can the GRADE approach be adapted to assess the certainty of a body of evidence that includes the results of modelling and to formulate recommendations? Based on findings from a web-based expert survey, a rapid literature review to identify criteria for assessing the “quality” of mathematical modelling studies, and on discussions and presentations at a workshop on the topic that was held April 2016 in Geneva, the authors conclude that modelling studies should indeed routinely be considered in the process of developing WHO guidelines, particularly in the evaluation of public health programmes, long-term effectiveness or comparative effectiveness. As for other types of evidence taken into consideration, there should be a systematic and transparent approach to identifying existing models that may be relevant and the quality and credibility of models should be systematically assessed. Relatively few adaptations are needed in the Grading of Recommendations Assessment, Development and Evaluation (GRADE) approach to rate the certainty of a body of evidence and to produce information that is used by guideline panels to formulate recommendations, based on the balance of benefits and harms and other considerations.

 MINOR COMMENTS:

1. Recommendation 4 is “No single ‘one-size-fits-all’ approach is appropriate to assess the quality of modelling studies. Existing frameworks and checklists may be adapted to a set of modelling studies by adding or omitting questions. In some situations, the approach will need to be developed *de novo*.” I’d prefer to turn it around: based on existing frameworks and checklists, generic criteria can be developed to assess the quality of modelling studies, although – depending on the situation –questions may have to be added or omitted. I am not convinced that in some situations a completely new approach is needed, and this would also not be advisable. The authors should either delete the last statement, or explain under which circumstances such a new approach is needed, ideally illustrated with an example.
2. Recommendation 8 is “The certainty of the evidence for modelling studies should be assessed and presented separately in summaries of the evidence (GRADE evidence profiles), and classified as high, moderate, low, or very low certainty.” In the text, the authors state that RCTs start as high certainty and observational studies as low certainty, although this certainty score may be up- or down-rated based on detailed assessment of five dimensions. Is it possible to give an indication of where modelling studies would start, with a justification? If not, can the authors describe factors to be considered when determining the start class? 
3. The questionnaire of the online survey on the use of mathematical modelling in guidelines for public health decision making is included as Figure S1, which combines a series of screen shots. The quality of this figure is poor and I recommend to include the questionnaire as a text document.

I confirm that I have read this submission and believe that I have an appropriate level of expertise to confirm that it is of an acceptable scientific standard.

# Response

**Published:** 2018-02-13

**Authors:** Susan Norris

**Affiliations:** World Health Organization, Switzerland

**Reviewer 1: Wilma A. Stolk, Erasmus MC, Department of Public Health, University Medical Center Rotterdam, Rotterdam, Netherlands**

Approved

Authors’ response: Thank you for reviewing and approving our paper. See below our responses to your comments. We have made changes in the text via tracked changes.

In this opinion article, the authors discuss when and how to incorporate the results of modelling studies into WHO guidelines, by addressing three questions: (1) When is it appropriate to consider modelling studies as part of the evidence that supports a guideline? (2) How should the quality and risk of bias in mathematical modelling studies be assessed? (3) How can the GRADE approach be adapted to assess the certainty of a body of evidence that includes the results of modelling and to formulate recommendations? Based on findings from a web-based expert survey, a rapid literature review to identify criteria for assessing the “quality” of mathematical modelling studies, and on discussions and presentations at a workshop on the topic that was held April 2016 in Geneva, the authors conclude that modelling studies should indeed routinely be considered in the process of developing WHO guidelines, particularly in the evaluation of public health programmes, long-term effectiveness or comparative effectiveness. As for other types of evidence taken into consideration, there should be a systematic and transparent approach to identifying existing models that may be relevant and the quality and credibility of models should be systematically assessed. Relatively few adaptations are needed in the Grading of Recommendations Assessment, Development and Evaluation (GRADE) approach to rate the certainty of a body of evidence and to produce information that is used by guideline panels to formulate recommendations, based on the balance of benefits and harms and other considerations.

Authors’ response: Thank you. This is a nice summary of our paper.

MINOR COMMENTS:

1. Recommendation 4 is “No single ‘one-size-fits-all’ approach is appropriate to assess the quality of modelling studies. Existing frameworks and checklists may be adapted to a set of modelling studies by adding or omitting questions. In some situations, the approach will need to be developed *de novo*.” I’d prefer to turn it around: based on existing frameworks and checklists, generic criteria can be developed to assess the quality of modelling studies, although – depending on the situation –questions may have to be added or omitted. I am not convinced that in some situations a completely new approach is needed, and this would also not be advisable. The authors should either delete the last statement, or explain under which circumstances such a new approach is needed, ideally illustrated with an example.

Authors’ response: Thank you. We agree and have deleted the last statement on page 9.

1. Recommendation 8 is “The certainty of the evidence for modelling studies should be assessed and presented separately in summaries of the evidence (GRADE evidence profiles), and classified as high, moderate, low, or very low certainty.” In the text, the authors state that RCTs start as high certainty and observational studies as low certainty, although this certainty score may be up- or down-rated based on detailed assessment of five dimensions. Is it possible to give an indication of where modelling studies would start, with a justification? If not, can the authors describe factors to be considered when determining the start class? 

Authors’ response: Thank you, we have addressed this issue as follows on page 9:

“We propose that within the GRADE system, modelling studies start at low certainty, and it is then possible to increase or decrease the certainty of modelling studies based on a set of criteria. The development of these criteria was beyond the scope of this article; a GRADE working group is addressing this issue ( http://www.gradeworkinggroup.org/).“ 

1. The questionnaire of the online survey on the use of mathematical modelling in guidelines for public health decision making is included as Figure S1, which combines a series of screen shots. The quality of this figure is poor and I recommend to include the questionnaire as a text document.

Authors’ response: Thank you. There is no text document for the survey but we have enlarged the screen shots to increase their readability (pages 22-27).
